# Pharmaceutical polymorph control in a drug-mimetic supramolecular gel†
†Electronic supplementary information (ESI) available: Synthetic and crystallographic experimental details, rheology, full crystallization and calculation details. See DOI: 10.1039/c6sc04126d



**DOI:** 10.1039/c6sc04126d

**Published:** 2016-10-07

**Authors:** Jonathan A. Foster, Krishna K. Damodaran, Antoine Maurin, Graeme M. Day, Hugh P. G. Thompson, Gary J. Cameron, Jenifer Cuesta Bernal, Jonathan W. Steed

**Affiliations:** a Department of Chemistry , University of Sheffield , Sheffield , S3 7HF , UK; b Department of Chemistry , Science Institute , University of Iceland , Dunhagi 3 , 107 Reykjavík , Iceland; c Department of Chemistry , Durham University , South Road , Durham , DH1 3LE , UK . Email: jon.steed@durham.ac.uk; d School of Chemistry , University of Southampton , Highfield , Southampton , SO17 1BJ , UK . Email: G.M.Day@soton.ac.uk; e Department of Chemistry , University of Cambridge , Cambridge , CB2 1EW , UK

## Abstract

We report the synthesis of a bis(urea) gelator designed to specifically mimic the chemical structure of the highly polymorphic drug substance ROY. Crystallization of ROY from toluene gels of this gelator results in the formation of the metastable red form instead of the thermodynamic yellow polymorph. In contrast, all other gels and solution control experiments give the yellow form. Conformational and crystal structure prediction methods have been used to propose the structure of the gel and show that the templation of the red form by the targeted gel results from conformational matching of the gelator to the ROY substrate coupled with overgrowth of ROY onto the local periodic structure of the gel fibres.

## Abstract 2

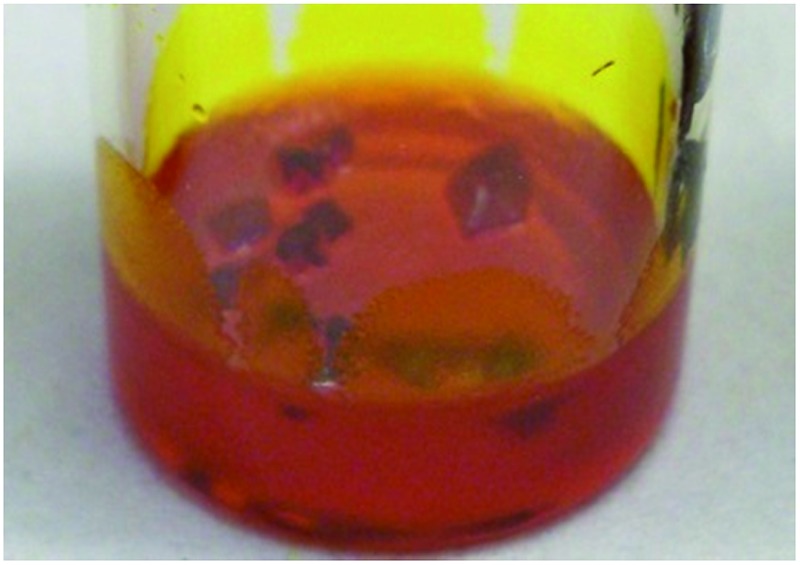
A supramolecular gel designed to chemically mimic the structure of a pharmaceutical compound controls the polymorphic outcome of the crystallization of the substrate.

## Introduction

The control of the solid state properties of crystalline drugs is of tremendous importance to the pharmaceutical industry. Active ingredient polymorphic form, particle size and crystal morphology profoundly influence the material's solubility, compressibility, friability, melting point, hygroscopy, bulk density and dissolution rate.^1–3^ Polymorph control also offers scope to transform an amorphous or hard-to-crystallise active pharmaceutical ingredient (API) into a readily handled, stable crystalline solid and is vital in obtaining regulatory approval.^4^ Examples of drug substances in which late-appearing or slow to nucleate polymorphs (as in the case of ritonavir^5^ or clopidogrel^6^) show that it can be very difficult to ensure that all possible crystal forms have been discovered. Moreover reliable identification and characterisation of polymorphic forms early in development can avoid lengthy and costly legal disputes as in the cefdinir case.^7^

In addition to careful removal of possible contaminating ‘seeds’ and highly controlled, reproducible crystallization conditions,^8^ advanced crystallization techniques such as crystallization from microemulsion droplets can in some cases reliably and selectively nucleate particular solid forms such as the thermodynamic form under ambient conditions.^9^ However there remains a significant need for solid form screening techniques that can target hard-to-nucleate polymorphs.

Crystallization in polymer hydrogels (*e.g.* agar, silica gel) of inorganic materials such as calcium carbonate^10–14^ and of biomolecules such as proteins is a well-known technique in which the gel limits convection and prevents sedimentation, allowing continuous, diffusion-limited growth^15^ and spatial control of nucleation.^16^ The gel environment can influence a number of factors such as crystal habit, polymorphism and enantiomorphism.^17–21^ Hydrogels^22^ have also been used to crystallise pharmaceuticals such a modafinil^23^ and the highly polymorphic model compounds ROY and carbamazepine have been crystallized within cubic polyethylene glycol diacrylate microgel particles.^18^

We have reported a novel polymorph discovery technique involving drug crystal growth in supramolecular organogels.^24–26^ Supramolecular gels offer a number of potential advantages over traditional polymeric hydrogels including the diverse range of functional groups that can be incorporated, the wide range of solvents gelled and the ability to redissolve the gels in order to recover the crystals. There have been a few recent reports of crystallization within low molecular weight supramolecular gels,^21^ notably work by Estroff on calcite crystallization in a bis(urea) gel,^10^ work by Gunnlaugsson on salt nanowires^27^ and work by Sanchez involving crystallization of aspirin, caffeine, indomethacin and carbamazepine in toluene-based tetraamide organogels^28^ and in lysine-based dendrons.^20^ In none of this work is there any suggestion of the gelators being designed to mimic the crystallization substrate, although carboxylates have been suggested to mimic carbonate in calcium carbonate hydrogel crystallizations.^29^ As a result the gel and crystal self-assembly are essentially orthogonal or only very weakly coupled and any differences in polymorphism observed serendipitous.^21,30^

We hypothesised that incorporating molecular features into a gelator that mimic those of the growing crystal would increase the probability of influencing crystal growth. In the present work we report the design of targeted bis(urea) gelators that gel to give a fibre surface that chemically mimics a target model drug substance, ROY,^31^ and offers the possibility of epitaxial crystal overgrowth and hence templation of metastable or hard-to-nucleate solid forms in a bespoke, drug-specific manner.

ROY was first synthesised by Eli Lilly as a precursor to olanzapine, a schizophrenia drug.^32^ There are at least ten crystal forms of ROY of which seven have been crystallographically characterised and are kinetically stable enough to be studied under near-ambient conditions.^33^ In fact, a crystal structure prediction study of the ROY molecule has demonstrated that even further polymorphs might be possible.^34^ The colours of the ROY polymorphs originate from conformational isomerism and allow for relatively facile *in situ* monitoring, with the yellow prism form (Y) being the most stable under ambient conditions.^35^ The substance also exhibits piezochromism.^36^ ROY represents a particularly suitable model system because of its large diversity of polymorphs, difficulty in controlling the crystallization outcome because of seeding effects and concomitant polymorphism, and its conformational polymorphism^37^ which offers the possibility of conformational matching with a targeted gel. Indeed one ROY polymorph has already been discovered by epitaxial nucleation.^32^

## Results and discussion

**Scheme 1:**
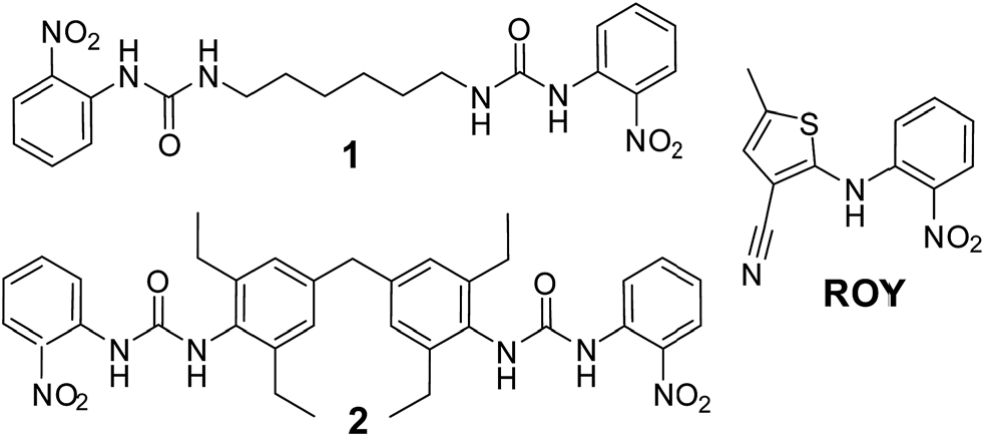
ROY and ROY-mimetic bis(urea) gelators **1** and **2**.

We have designed a series of gelators incorporating *o*-nitroaniline-derived functional groups, mimicking the *o*-nitroaniline substituent in ROY, grafted onto a variety of bis(urea) gel-forming cores. We anticipate that these targeted gelators will self-assemble to give gels^38,39^ in which the surface of the gel fibre consists of a locally ordered array of *o*-nitroaniline-derived functional groups, closely matching the *o*-nitroaniline substituent in ROY. The series of bis(urea) compounds were readily prepared from the reaction of *o*-nitrophenylisocyanate with five different diamine cores (Scheme 1 and see ESI, Scheme S1†). The compounds were tested for gelation in a variety of solvents and compounds **1** and **2** were found to be effective gelators, whereas the other three compounds failed to gel the majority of the solvents tested and were not further investigated (see ESI†). While bis(ureas) commonly give high aspect ratio solid particles, the evolution of these fibrillar materials into gels is subject to solubility constraints and a subtle balance of interactions that are not currently fully understood.^40–42^

Compound **1** formed gels at 1% weight to volume in almost all solvents studied (acetonitrile, methanol, ethanol, acetone, dichloromethane, chloroform, ethyl acetate and toluene). Gels were not observed in water or THF. However, the gels are opaque and fragile, breaking apart to form a precipitate if gently shaken. The chloroform, toluene, acetonitrile and acetone gels are unstable and form a precipitate after a number of days whilst gels from other solvents remain stable. The opacity of these gels renders them unsuitable for crystallization studies and as a result efforts concentrated on compound **2**.

**Fig. 1:**
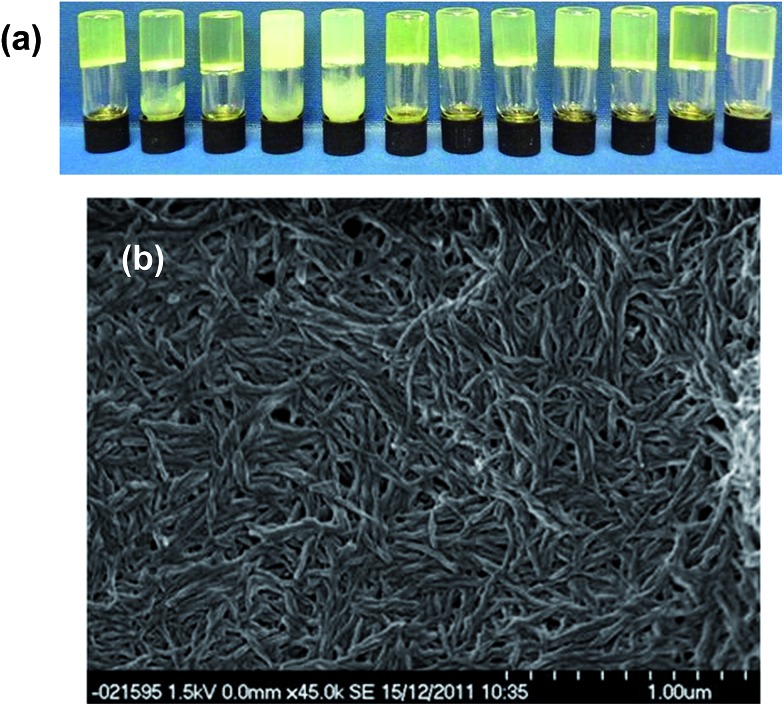
(a) Organogels formed by **2** in (left to right) dichloromethane, chlorobenzene, 1,2-dichlorobenzene, benzene, toluene, acetonitrile, methanol, ethanol, 1-propanol, 1-butanol, nitrobenzene and ethyl acetate (b) SEM micrograph of the toluene xerogel of **2** at 1% w/v.

Compound **2** forms robust, stable, translucent gels in a wide range of solvents (see ESI†) including acetonitrile, methanol, acetone, ethyl acetate and toluene as shown in Fig. 1. Compound **2** is much less soluble than **1** failing to dissolve fully in a number of the solvents at 1% w/v. Undissolved material tends to inhibit gel formation and the use of lower concentrations of gelator results in more translucent and homogeneous gels. SEM studies on the xerogel show an entangled network of fine fibres. The translucent appearance of the gels makes them highly suited to crystallization studies. Moreover the fact that this gelator can gel a range of organic solvents allows a great deal of scope to co-dissolve the gelator with drug substances of varying solubility.^24^

Solutions containing 100 mg mL^–1^ of ROY were crystallised by slow cooling from toluene gels of the designer gelator **2**, as well as under the same conditions from toluene control solutions containing either no gelator, or one of four different bis(urea) gelators (**3–6**) with no structural similarity to ROY. These non-specific gelators contained substituents derived from l-alanine (**3**),^43^ l-phenylalanine (**4**),^44^ l-lysine (**5**) and triethoxysilane (**6**)^45^ instead of the ROY-mimetic nitrophenylanaline-derived substituent (see ESI† for gelator structures). A further gelator with a l-phenylalanine substituent and the same diphenylmethane derived spacer as **2** (compound **7**) was also prepared. Toluene was selected as the solvent because a wide variety of the gelators reliably form gels in the solvent without sonication. Samples were heated in sealed vials until all material was dissolved and allowed to cool to room temperature on the bench top.

**Fig. 2:**
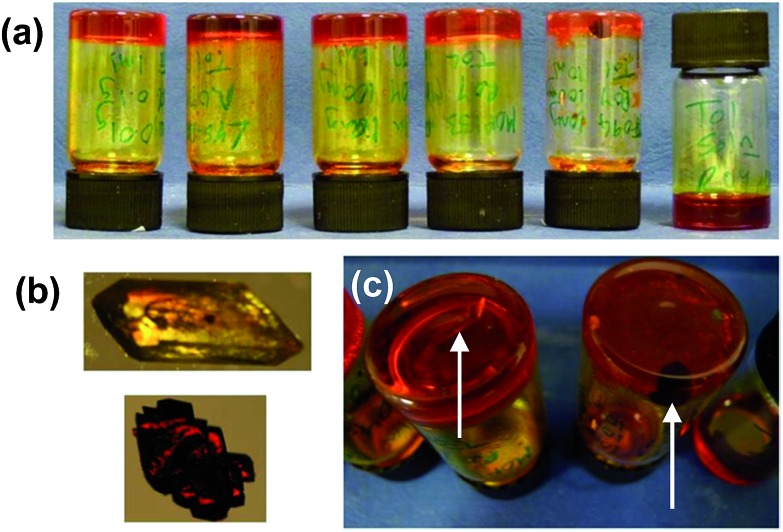
(a) Crystals of ROY grown from four non-specific control gels and from gels of **2** (left to right: **3–6** and **2**) and a solution phase control experiment. (b) Isolated gel-grown crystals of the Y and R forms. (c) Y-Form crystals growing in a toluene gel of non-specific gelator **6** (left) and R-form crystals growing in toluene gel of **2**, (right; arrows point to individual crystals).

After leaving the samples for one month all of the non-specific generic gelators and the solution control experiment produced large yellow blocks identified by single crystal X-ray unit cell determination, IR spectroscopy and XRPD (see ESI Fig. S1 and S2†) as the thermodynamically most stable monoclinic Y form. Under the same conditions, 1% w/v gels of **2** produced red crystals corresponding to the metastable, triclinic red (R) form, also characterised by unit cell determination, IR and XRPD. Fig. 2 shows images of the crystals obtained from different gels. These results indicate that the designer gelator, **2**, induces the crystallisation of a different polymorph of ROY to that obtained from solution or from a range of gels with no structural similarity to ROY.

In order to test the generality and reproducibility of this observation, crystallizations of ROY in toluene at concentrations 50–200 mg mL^–1^ were undertaken from gels of ROY mimic **2**. The outcome of these experiments were compared with samples crystallized from solution and from four different non-specific gelators bearing either amino acid substituents (**3**, **4** and **7**) or triethoxysilane terminal groups (**6**) as well as different spacer units between the urea functionalities. Gels were formed with 1% w/v of gelator in each case except for compound **3**, which was used at 1.5% w/v. An additional sample containing a non-gelling solution saturated with **2** at room temperature was also investigated. The purpose of this reference was to test whether any differences observed were due to the gel state or compound **2** acting as a solution-based crystallization additive.

**Fig. 3:**
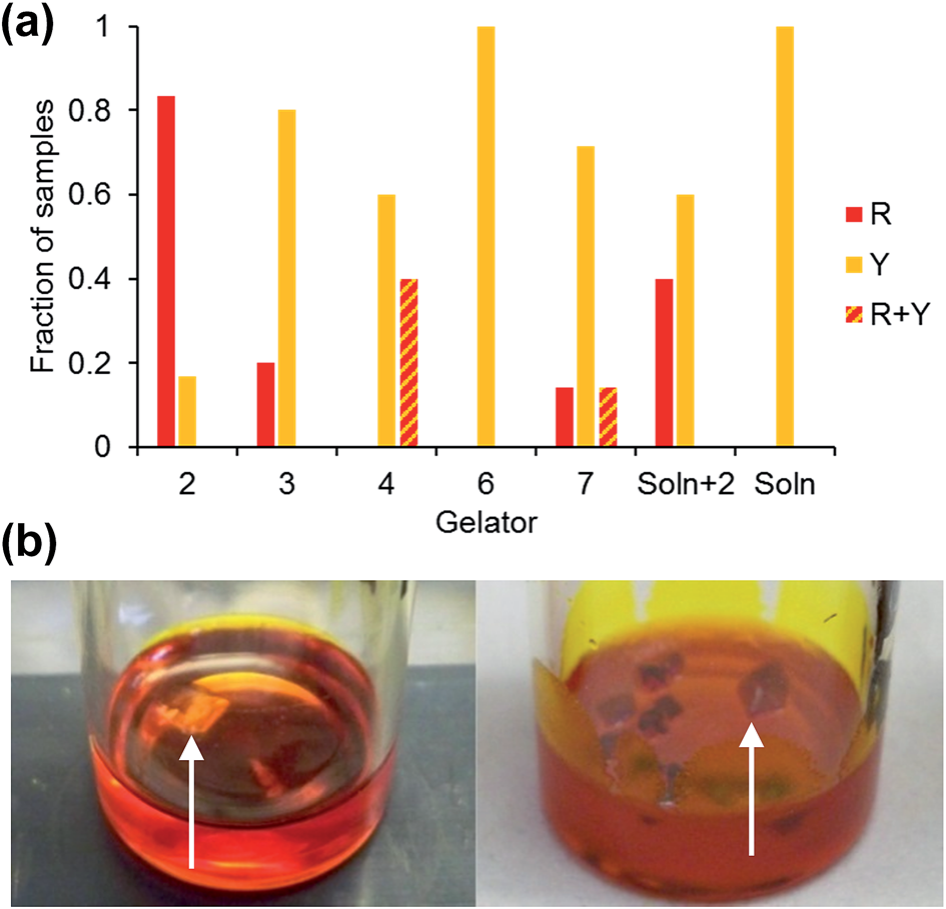
(a) Collated data comparing the form of ROY obtained from 100 mg mL^–1^ toluene gels of designer gelator **2**, non-specific gelators **3**, **4**, **6** and **7**, from toluene solution saturated with **2** and from solution. R + Y denotes concomitant crystallisation of both the R and Y crystal forms in the same sample. (b) Crystallization of the Y form of ROY from a toluene gel of control compound **7** and the R form from a toluene gel of **2** (arrows point to individual crystals).

The crystallisations were repeated in a series of experiments between 5 and 12 times and the results detailed in ESI Tables 1 and 2† and the collated results for samples loaded with 100 mg mL^–1^ ROY are summarised in Fig. 3. The optimised experimental setup involved addition of 1 mL toluene to the gelator (10 mg) and ROY (100 mg) in a vial, which was then sealed and heated to 140 °C to avoid heteroseeding. A DrySyn multi-reaction station was used to in order to achieve a consistent, controlled cooling profile. Crystallisation generally took place over several hours to weeks. Clear differences in crystal colour and shape allow the different polymorphs to be distinguished. Solid forms were confirmed by IR spectrometry and XRPD analysis. Analyses of the crystals revealed two different polymorphs identified as the Y and R forms,^46^ sometimes appearing concomitantly. All the crystals formed were stable and did not undergo any phase transition *in situ* after several months.

Gels of **2** loaded with 100 mg mL^–1^ ROY yielded the metastable R form is almost every case, with only two of the twelve repeats giving the Y form. These two anomalous results are attributed to accidental heteroseeding with Y particles. In contrast, the vast majority of samples from the control gelators produced the Y form (which is the most thermodynamically stable under ambient conditions). The control experiments in toluene devoid of any gelator also resulted in the thermodynamic Y form. Gelator **3** produced four Y and one R samples out of five whilst the remaining one gave a concomitant mixed R/Y sample. Gelator **4** gave one concomitant R/Y sample, with one sample transforming to Y after three days and the remaining six yielded Y crystals. Gelator **6** gave only Y crystals. Gelator **7**, which has the same spacer between the bis(urea) but a phenylalanine derived end group unrelated to ROY, and therefore potentially provides the best comparison, gave the Y form in five repeats whilst one gave the R form and one a mixture of the R and Y forms.

Samples crystallised at lower concentrations of ROY (50 mg mL^–1^) typically took longer to crystallise and the R form was only observed from gels of **2** with all other samples giving the Y form. In contrast, at 200 mg mL^–1^ of ROY, only the Y form was observed in gels of **2** indicating high concentrations may diminish the gel's selectivity.

The solution controls only gave the Y form (18 repeats). The Y form was also obtained in three out of five crystallizations from solutions of gelator **2** at a concentration too low to result in gel formation. This suggests that compound **2** has only a small effect on crystal growth as a solution based additive and it is the solid fibres of gels of **2** that induce formation of the R form.

On balance this screen suggests that the designer gels of **2** strongly bias ROY crystallization towards formation of the metastable triclinic red R form. The difficulty in controlling ROY polymorphic outcome from solution is well documented.^31,33^ In one study a solution of ROY evaporated from 10 000 500 μm gold islands on a single plate produced six out of the seven stable forms of ROY.^47^ An additional factor is that in some samples the crystals grow against the sides of the vials and on the surface of the gels. In these cases heteronucleation on the glass vial or from dust at the gel surface may determine the crystal form rather than the influence of the gel matrix. The microscopic seeding of the Y form is also a potential confounding factor. In this context, the results are remarkably clear-cut indicating that the ROY-mimetic nitrophenylaniline substituent exerts a clear influence on the crystallization outcome.

In order to understand the mechanism by which gels of **2** consistently produce a different polymorphic outcome in the crystallization of ROY compared to other bis(urea) gels and solution control experiments we compared the structure of **2** with the crystal structures of the R and Y forms of ROY. The tendency of **2** to form highly anisotropic gel fibres means it is not possible to characterise **2** by single crystal X-ray diffraction and powder diffraction gives broad, poorly defined peaks (see ESI†). We therefore applied computational structure prediction methods to investigate the molecular geometry of **2**. The conformational flexibility of **2** means that structural determination in this way remains highly challenging.

**Fig. 4:**
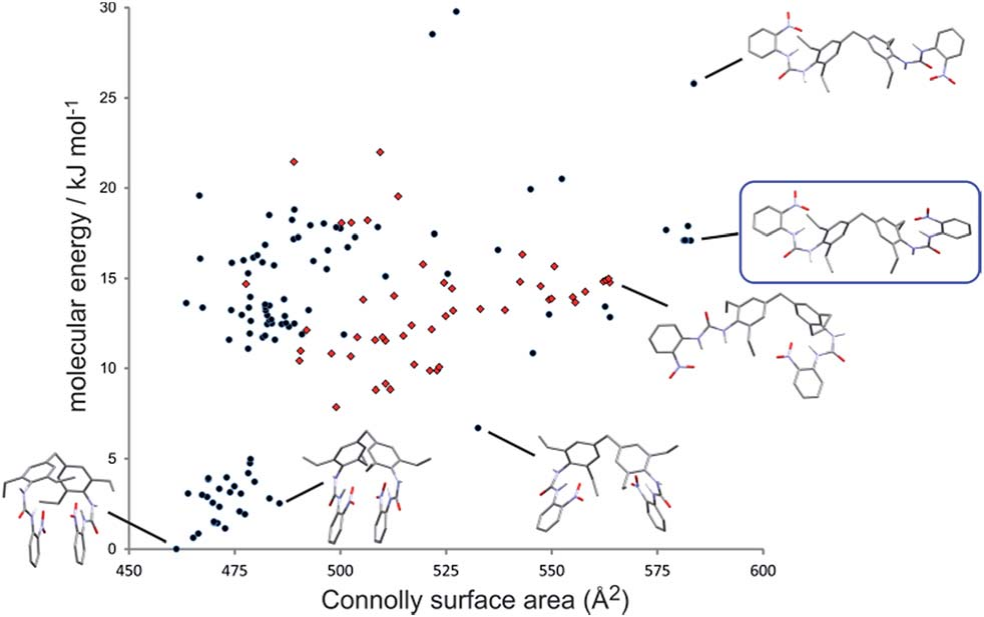
Conformational landscape of gelator **2**. Each point represents the calculated (DFT-D) energy and Connolly surface area of a predicted conformer. Selected conformers are shown, with all hydrogen atoms hidden for clarity, apart from the urea hydrogens. Red points show conformations with one urea group in the *anti*–*anti* conformation. No conformations with both ureas in the *anti*–*anti* conformation are found in this energy range. The predicted most likely conformation, as a balance of intramolecular energy and extendedness, is enclosed in a blue box.

The conformational landscape of **2** was predicted using force field based searches, using the OPLS-AA force field within a low-mode conformational search,^48^ followed by dispersion-corrected density functional theory (DFT-D) molecular geometry optimization. These searches found a large number of possible conformers, the lowest energy of which adopt a compact geometry in which nitroaniline groups on each end of the molecule are folded together. However, a recent computational study^49^ has demonstrated that flexible molecules preferentially adopt higher energy, extended conformers in the solid state, which enables greater intermolecular interactions. We calculate the Connolly surface area of all structures as a measure of the extendedness of the conformer (Fig. 4). Many extended conformers are available within the relevant energy range for conformers in solids (approximately 25 kJ mol^–1^),^49^ which open the nitroaniline groups to a more accessible arrangement. Although we cannot select one of the predicted conformers as that which forms the gel, we propose that the fibres are composed of one of these extended conformers of **2**. As a predictor of which conformer is most likely, it has been suggested that a biasing term based on the surface area is added to the DFT-D conformational energies to approximate the increased stabilizing intermolecular interactions available to extended conformations.^49^ The preferred conformer with this term included is shown in the blue box in Fig. 4.

Interestingly, few of the candidate conformers of **2** exhibited the urea conformation that is required to form the common urea α-tape type of packing mode based on the ubiquitous R12(6) hydrogen bonded ring geometry.^39,50,51^ A relatively small number of predicted conformers have one of the urea groups in an *anti*–*anti* conformation (Fig. 4), where both hydrogen atoms are oriented *anti* to the carbonyl oxygen. The lowest energy conformer with both ureas in the *anti*–*anti* conformation is found 85 kJ mol^–1^ above the lowest energy conformer (off the scale of Fig. 4). These results suggest that it is unlikely that the gel fibres form as a consequence of strong uni-directional hydrogen bond tapes.

Intramolecular hydrogen bonds from the urea to nitro groups are present in all low energy conformers of **2**, forming 6-membered rings which would be predicted by Etter's hydrogen bonding rules.^52^ These intramolecular hydrogen bonds might be expected to interfere with intermolecular hydrogen bonding.

**Fig. 5:**
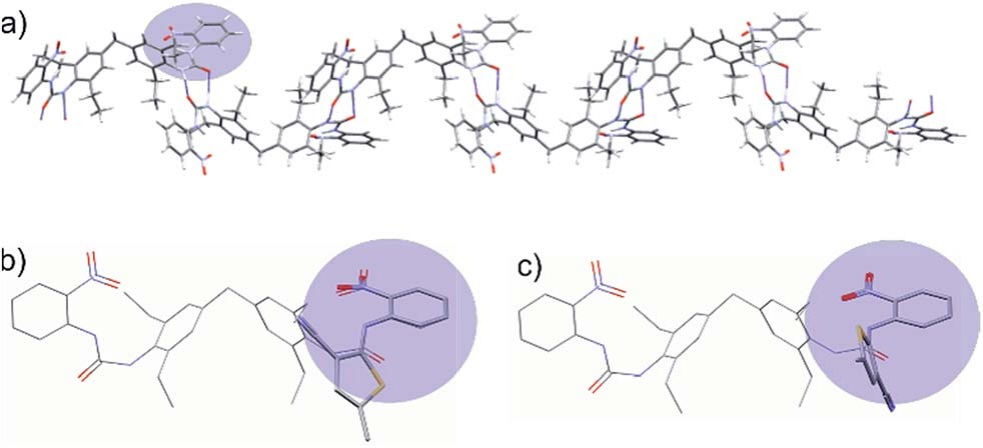
(a) Hydrogen bond chains in the lowest energy predicted crystal structure resulting from an extended conformer of **2**. Hydrogen bonds are indicated as thin blue lines. The conformer leading to this structure is enclosed in a blue box in Fig. 4. (b) Overlay of the extended conformer of **2** with the ROY conformation from the R polymorph, showing a good steric match of the nitroaniline group to ROY. (c) Overlay of the extended conformer of **2** with the ROY conformation from the Y polymorph. The thiophene rings and urea are nearly at right angles, showing a poor steric match.

To explore the solid state packing of **2**, crystal structure prediction (CSP) calculations were performed on a selection of the lowest energy and most extended predicted conformers. Previous work has shown that CSP methods designed to predict crystal structure can help understand the molecular arrangement in gel fibres.^53–55^ The CSP calculations involved a quasi-random search^56^ for structures in a set of commonly observed space groups, followed by lattice energy minimization with the Crystal Optimizer^57^ and DMACRYS^58^ software, using an atomic multipole based atom–atom force field. Most of the lowest energy predicted crystal structures from both the folded and extended molecular conformers contained R22(8) NH···O=C hydrogen bond rings involving the non-intramolecularly hydrogen bonded urea hydrogen. These dimeric interactions at each end of the molecule result in infinite chains (Fig. 5a), which would be expected to lead to fast growth in the direction of the chain.

We then attempted to correlate the CSP results with the experimental XRPD pattern obtained from xerogels of **2**. While XRPD data for xerogels is generally broad and featureless because of the lack of long range order in gel fibres, we obtained similar XPRD patterns from xerogels of **2** from a range of solvents suggesting that gels of **2** adopt a similar structure regardless of solvent. The xerogel XRPD data did not prove to be a match for any of the calculated structures involving the folded conformer, however the XRPD patterns corresponding to the lowest energy calculated structures of the extended conformers possessed considerable similarity to the experimental xerogel XRPD data (see ESI†). Hence there is justification for regarding packing features of the lowest energy calculated crystal structures of the extended conformer of **2**, and particularly the hydrogen bond chains of molecules, as a model for the way in which compound **2** packs in the gel fibrils.

The intramolecular hydrogen bonding maintains planar nitroaniline units at either end of the gelator molecule, which extend outwards from the hydrogen bonded chains of molecules (Fig. 5a). Therefore, the nitroaniline would be expected to be exposed on the surface of gel fibres, thus being available for interaction with ROY molecules. Significant differences between ROY polymorphs lie in the dihedral angle between the phenyl and thiophene rings, and it is these conformational differences that are responsible for the distinctive colours of the different solid forms. Fig. 5 shows an overlay of the molecular structure of **2** taken from this calculated structure with the molecular structures of ROY observed in the Y and R experimental crystal structures, matching the nitrophenyl groups in the two molecules. The thiophene orientation in the R conformation gives a close steric match to the urea in **2** and aligns the polar thiophene sulfur with the urea oxygen (Fig. 5b). In contrast, the Y conformation places the thiophene at right angles to the urea group in **2** (Fig. 5c). Thus, **2** gives a better steric and electrostatic match to the R than the Y conformation of ROY. This is a result of the intramolecular hydrogen bond, present in all low energy conformers of **2**, which strongly favours the nearly-coplanar arrangement of urea and nitrophenyl groups. In the proposed structure of **2**, these groups are presented periodically on the surface of the gel fibres allowing them to interact with the growing nucleus. We thus hypothesise that the ability of gels of **2** to template the R form from a supersaturated solution of ROY arises from a match of the R conformer with the periodic structure of the ROY-mimetic portion of the gelator resulting in epitaxial overgrowth of this metastable form. The effect of conformational matching between the gelator and ROY for R would be weakened if the gelator conformation was flexible and likely to be dynamic at the surface of the gel fibre. However, conformational dynamics of the nitroaniline group are expected to be minimised by the intramolecular hydrogen bond and the rigidity of the urea group. The other control gels are likely to adopt the more conventional urea α-tape type packing and do not possess chemical functionality that can interact with ROY in supersaturated solution. As a result, the polymorphic outcome is the same as the solution control experiments.

## Conclusions

In conclusion, organogels of a specifically targeted gelator that mimics the functional groups of the highly conformationally polymorphic substrate ROY reproducibly results in the crystallization of the metastable R polymorph of ROY. Under identical conditions, crystallization from generic gels, from solution and from solutions containing the designer gelator at sub-critical gelation concentration all give the thermodynamic Y form. The likely structure of the designer gelator **2** was calculated using conformation and crystal structure prediction methodologies to give insight into the structure matching between gel and the ROY forms. The unique effect of designer gels of **2** is postulated to arise from conformational matching with the pendant ROY-mimetic functional groups on the gel fibre surface, coupled with the local periodicity of the gel fibre allowing heteronucleation of the R form. This study demonstrates the potential of designer supramolecular gels to be used in a targeted way to influence the polymorphism of pharmaceutical compounds.
