# Supplementary material for: Pharmaceutical polymorph control in a drug-mimetic supramolecular gel
Source: Chem Sci. 2016 Oct 7;8(1):78–84. doi: 10.1039/c6sc04126d (PMC5304659; doi:10.1039/c6sc04126d)
Supplement: Supplementary file 1 [file SC-008-C6SC04126D-s001.pdf]

Electronic Supplementary Material (ESI) for Chemical Science.
This journal is © The Royal Society of Chemistry 2016

Pharmaceutical Polymorph Control in a Drug-Specific Designer Supramolecular Gel

Jonathan A. Foster,[a] Krishna K. Damodaran,[b] Antoine Maurin,[c] Graeme M. Day*[d], Hugh P. G. Thompson,[e] Gary J. Cameron,[c] Jenifer Cuesta Bernal[c] and Jonathan W. Steed*[c]

a) *Department of Chemistry, University of Sheffield, Sheffield, S3 7HF, UK.*
b) *Department of Chemistry, Science Institute, University of Iceland, Dunhagi 3, 107 Reykjavík, Iceland.*
c) *Department of Chemistry, Durham University, South Road, Durham, DH1 3LE, UK E-mail: jon.steed@durham.ac.uk.*
d) *School of Chemistry, University of Southampton, Highfield, Southampton, SO17 1BJ, UK. E-mail: G.M.Day@soton.ac.uk*
e) *Department of Chemistry, University of Cambridge, Cambridge, CB2 1EW, UK*

Supplementary Material

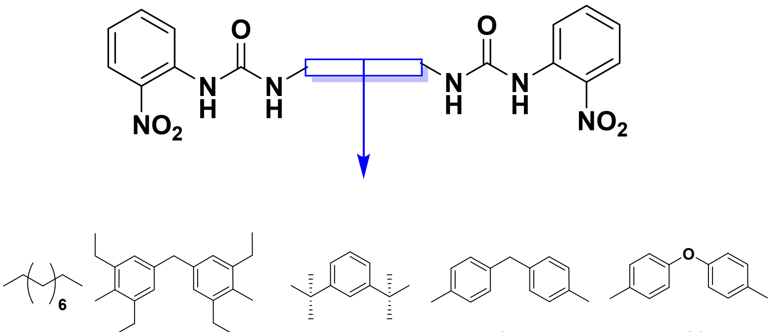

1 2 8 9 10

Scheme S1 ROY-mimetic bis(urea) compounds prepared in this work.

Synthesis

1,3-Bis(α-aminoisopropyl)benzene was synthesied following a reported procedure (L. Dahlenburg, H. Treffert, F, W. Heinemann, *Inorg Chim Acta* **2008***, 361*, 1311).

1-(2-nitrophenyl)-3-[6-[(2-nitrophenyl)carbamoylamino]hexyl]urea (**1**)

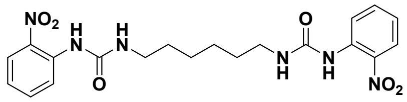

1,6diamino hexane (0.35 g, 3.046 mmol) dissolved in chloroform (20 mL) was added dropwise to a solution of 2-nitrophenyl isocyanate (1 g, 6.093 mmol) in chloroform (20 mL). A bright yellow precipitate rapidly formed from the solution which was stirred for a further hour at room temperature. The precipitate was filtered, washed with DCM and dried in a heating pistol for 10 minutes to give the product as a bright yellow solid (1.3 g, 2.99 mmol, 84%). ^{1}H NMR (600MHz, DMSO-d$_6$, *J*/Hz): 9.30 (2 H, s, ArNH), 8.30 (2 H, dd, *J* 8.6, 1.3, ArH), 8.02 (2 H, dd, *J* 8.6, 1.3, ArH), 7.60 (2 H, ddd, *J* 8.7, 7.2, 1.6, ArH), 7.48 (2 H, t, *J* 5.0, NH), 7.08 (2 H, ddd, *J* 8.4, 7.2, 1.3, ArH), 3.08 (4 H, td, *J* 6.9, 5.4, NHCH$_2$), 1.44 (4 H, p, *J* 6.7, NHCH$_2$CH$_2$), 1.36 – 1.27 (4 H, m, CH$_2$CH$_2$CH$_2$). ^{13}C{^{1}H} NMR (151 MHz, DMSO-d$_6$): 154.63 (s, CO), 137.09 (s, ArC), 136.47 (s, ArC), 135.39 (s, ArC), 125.72 (s, ArH), 122.30 (s, ArC), 121.64 (s, ArC), 40.87-39.60 (s, under DMSO multiplet, NHCH$_2$), 29.79 (s, NHCH$_2$CH$_2$), 26.56 (s, CH$_2$CH$_2$CH$_2$). Anal. calc. for $C_{20}H_{42}N_4O_6$: C, 54.05; H, 5.44; N, 18.9. Found: C, 52.59; H 5.24; N: 13.19 %.

1-[4-[[3,5-diethyl-4-[(2-nitrophenyl)carbamoylamino]phenyl]methyl]-2,6-diethyl-phenyl]-3-(2-nitrophenyl)urea (**2**)

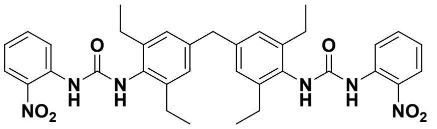

2-Nitrophenylisocyanate (2.5 g, 15.2 mmol) was added to a solution of 4,4-methylenebis(2,6 diethylaniline) (2.36 g, 7.6 mmol) in dry chloroform (400 mL) at room temperature. The resulting mixture was stirred under nitrogen and a gelatinous precipitate start appearing after 5 minutes. The mixture was then heated under reflux for 18 hours and cooled to room temperature, filtered, washed with chloroform (4 x 50 mL) and dried. The yellow precipitate was then triturated with 200 ml chloroform for 24h and then filtered, washed with chloroform (100 ml) and dried. The solid was ground to give **2** as a fine pale yellow powder (3.9 g, 6.1 mmol, 80 %). ^{1}H NMR (400 MHz, DMSO-d_6) δ = 9.70 (s, 2H, $NO_2ArN\underline{H}$), 8.83 (s, 2H, $ArN\underline{H}$), 8.32 (d, *J*=8.5, 2H, $NO_2Ar\underline{H}$), 8.08 (d, *J*=8.4, 2H, $NO_2Ar\underline{H}$), 7.66 (t, *J*=8.3, 2H, $NO_2Ar\underline{H}$), 7.31 – 6.75 (m, 6H, 2 $NO_2Ar\underline{H}$ and 4 $Ar\underline{H}$), 3.92 (d, *J*=32.8, 2H, $ArC\underline{H}_2Ar$), 2.62 – 2.51 (m, 8H, $C\underline{H}_2CH_3$), 1.13 (t, *J*=7.3, 12H, $CH_2C\underline{H}_3$). ^{13}C NMR (101 MHz, DMSO-d_6) δ = 153.61 (s, $\underline{C}O$), 142.25 (s, $Ar\underline{C}$),, 140.32 (s, $Ar\underline{C}$), 137.63 (s, $Ar\underline{C}$), 136.14 (s, $Ar\underline{C}$), 135.46 , 131.82 (s, $Ar\underline{C}$), 126.82 (s, $Ar\underline{C}$), 125.83 (s, $Ar\underline{C}$), 122.36 (d, *J* = 40.3) (s, $Ar\underline{C}$), 24.90 (s, $\underline{C}H_2CH_3$), 15.19 (s, $CH_2\underline{C}H_3$). ESI-MS Calcd.(M^+) 638.7, Found 638.8. Anal. calc for $C_{35}H_{38}N_6O_6$: C, 65.82; H, 6.00; N, 13.16. Found: C, 65 .72; H, 5.97; N, 13.13 %

Compounds **8**, **9** and **10** were synthesised by following a general procedure. The diamine (1 equiv) was dissolved in dissolved in 200 mL dry chloroform and of 2-nitrophenyl isocyanate was added directly to the solution. The mixture was refluxed overnight under nitrogen atmosphere, cooled to room temperature and the yellow precipitate obtained was filtered, washed with chloroform and dried.

1-*[1-Methyl-1-(3-{2-methyl-2-[3-(2-nitrosophenyl)ureido]propyl}phenyl)ethyl]-3-(2-nitrosophenyl)urea* (**8**)

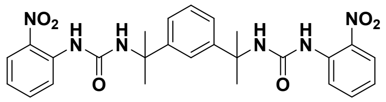

^{1}H NMR (400 MHz, DMSO-d_6) δ = 9.40 (s, 2H, $NO_2Ar\underline{H}$), 8.19 (dd, *J*=8.7, 1.3, 2H, $NO_2Ar\underline{H}$), 8.04 – 7.93 (m, 4H, $NO_2Ar\underline{H}$), 7.55 – 7.44 (m, 3H, 2 $NO_2Ar\underline{H}$ & 1$Ar\underline{H}$), 7.27 – 7.18 (m, 3H, 1$Ar\underline{H}$ & 2 $CN\underline{H}$), 7.05 (ddd, *J*=8.5, 7.1, 1.3, 2H, $Ar\underline{H}$), 1.60 (s, 12H, $ArC(C\underline{H}_3)_2$. ^{13}C NMR (101 MHz, DMSO-d_6) δ = 153.38 (s, CO), 148.09 (s, $Ar\underline{C}$), 136.98 (s, $Ar\underline{C}$), 136.43 (s, $Ar\underline{C}$), 135.22 (s, $Ar\underline{C}$), 128.04 (s, $Ar\underline{C}$), 125.66 (s, $Ar\underline{C}$), 122.92 (s, $Ar\underline{C}$), 122.42 (s, $Ar\underline{C}$), 121.58 (d, $Ar\underline{C}$, *J*=7.3), 55.42 $Ar\underline{C}(CH_3)_2$, 30.20 (s, $ArC(\underline{C}H_3)_2$). ESI-MS Calcd.(M^+) 520.55, Found 520.86. Anal. calc for $C_{26}H_{28}N_6O_6$: C, 59.99; H, 5.42; N, 16.14. Found: C, 59.56; H, 5.36; N, 15.96.

3-(2-Nitrophenyl)-1-[4-({4-[3-(2-nitrophenyl)ureido]phenyl}methyl)phenyl]urea (**9**)

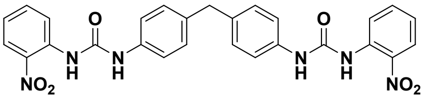

^{1}H NMR (400 MHz, DMSO-d_6) δ = 9.79 (s, 2H, $NO_2ArN\underline{H}$), 9.57 (s, 2H, $ArN\underline{H}$), 8.31 (dt, *J*=8.6, 1.3, 2H, $NO_2Ar\underline{H}$), 8.09 (dd, *J*=8.4, 1.5, 2H, $NO_2Ar\underline{H}$), 7.69 (ddd, *J*=8.7, 7.2, 1.6, 2H, $NO_2Ar\underline{H}$), 7.41 (d, *J*=8.3, 4H, 2 $NO_2Ar\underline{H}$ & 2 $Ar\underline{H}$), 7.27 – 7.10 (m, 6H, $Ar\underline{H}$), 3.84 (s, 2H, $ArC\underline{H}_2Ar$). ^{13}C NMR (101 MHz, DMSO-d_6) δ = 152.25 (s, $\underline{C}O$), 137.97 (s, $Ar\underline{C}$), 137.57, (s, $Ar\underline{C}$), 136.13(s, $Ar\underline{C}$), 135.46 (d, *J*=3.1, $Ar\underline{C}$), 129.47 (s, $Ar\underline{C}$), 125.84 (s, $Ar\underline{C}$), 122.92 (s, $Ar\underline{C}$), 122.58 (s, $Ar\underline{C}$), 119.27 (s, $Ar\underline{C}$), 57.95 – 29.12 (s, under DMSO multiplet, $Ar\underline{C}H_2Ar$). ESI-MS Calcd.(M^+) 526.51, Found 548.71 (M + Na^+) Anal. calc for $C_{27}H_{22}N_6O_6$: C, 61.59; H, 4.21; N, 15.96. Found: C, 61.16; H, 4.22; N, 15.77.

1-(3-Nitrophenyl)-3-(4-{4-[3-(o-nitrophenyl)ureido]phenoxy}phenyl)urea (**10**)

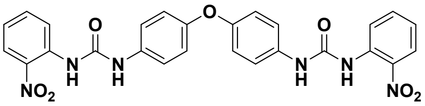

^{1}H NMR (400 MHz, DMSO-d_6) δ = 9.84 (s, 2H, $NO_2ArN\underline{H}$), 9.59 (s, 2H, $ArN\underline{H}$), 8.37 – 8.27 (m, 2H, $NO_2Ar\underline{H}$), 8.09 (dd, *J*=8.5, 1.6, 2H, $NO_2Ar\underline{H}$), 7.69 (ddd, *J*=8.7, 7.2, 1.6, 2H, $NO_2Ar\underline{H}$), 7.54 – 7.44 (m, 4H, 2 $NO_2Ar\underline{H}$ & 2 $Ar\underline{H}$), 7.19 (ddd, *J*=8.5, 7.2, 1.4, 2H, $Ar\underline{H}$), 7.03 – 6.92 (m, 4H, $Ar\underline{H}$). ^{13}C NMR (101 MHz, DMSO-d_6) δ = 152.66 (s, $\underline{C}O$), 152.31 (s, $Ar\underline{C}$), 137.95 (s, $Ar\underline{C}$), 135.47 (d, *J*=3.9, $Ar\underline{C}$), 135.09 (s, $Ar\underline{C}$), 125.85 (s, $Ar\underline{C}$), 122.89 (s, $Ar\underline{C}$), 122.58 (s, $Ar\underline{C}$), 120.82 (s, $Ar\underline{C}$), 119.34 (s, $Ar\underline{C}$). ESI-MS Calcd.(M^+) 528.48, Found 528.8. Anal. calc for $C_{26}H_{20}N_6O_7.H_2O$: C, 57.14; H, 4.06; N, 15.38. Found: C, 56.81; H, 3.89; N, 15.12.

Gel Preparation

Compounds **1, 2, 8, 9** and **10** were tested in a range of solvents for evidence of gel formation. The compound (0.01 g) was heated in 1 mL of solvent (1 % w/v) in a sealed vial until fully dissolved and then cooled to room temperature. After 24 h, gel formation was characterised by a simple vial inversion test; if the solvent was fully immobilised it was considered to have gelled.

Compound **8** and **9** failed to gel any of the solvent tested but compound **10** was able to form gel in nitromethane and nitrobenzene.

Compound **2** turned out to be an excellent gelator, able to form gel in the solvent listed below at 1 wt% or 0.5 wt% (starred): dichloromethane, chlorobenzene, *o*-xylene, *m*-xylene, *p*-xylene*, benzene, toluene, acetonitrile*, methanol*, ethanol*, 1-propanol*, 2-propanol*, 1-butanol*, 2-butanol*, 1-pentanol, nitromethane, nitrobenzene, ethyl acetate*.

Gel Characterisation

T_{gel} Characterisation of Compound 2

T_{gel} was measured by the drop ball method using a custom-made glass ball (0.25 g). In a typical experiment, the gelator (0.01 g) was heated in 1 mL of toluene (1 % w/v) in a sealed vial until fully dissolved. The solution was cooled to room temperature and after 12 hours the vial was opened and the glass ball was placed on the gel surface, sealed and gradually heated in an oil bath. The temperature at which the ball drops into the bottom of the vial was recorded as the gel dissociation temperature (T_{gel}) and was found to be 92 ^{o}C at 1 wt %. The minimum gel concentration (MGC) of **2** in toluene was found to be 0.0075g/mL

Rheology of Compound 2

Rheology experiments were performed using a TA Instruments Advanced Rheometer 2000. A concentric cylinder couette geometry (25 mm rough plate) with a gap of 2500 μm and 2mL of sample was used in each case. 0.02 g/mL of gelator in toluene (2 mL) was prepared in a glass vial, sealed and carefully heated until the gelator had fully dissolved. The hot gelator solution was transferred into the glass cylindrical mould (diameter 30 mm) on the rough plate, cooled to 20°C and equilibrated for 30 minutes. Oscillatory stress sweep measurement was performed over a range of 0.01-100 Pa with a constant frequency value of 1 Hz. In addition frequency sweep measurements were performed at 1% w/v.

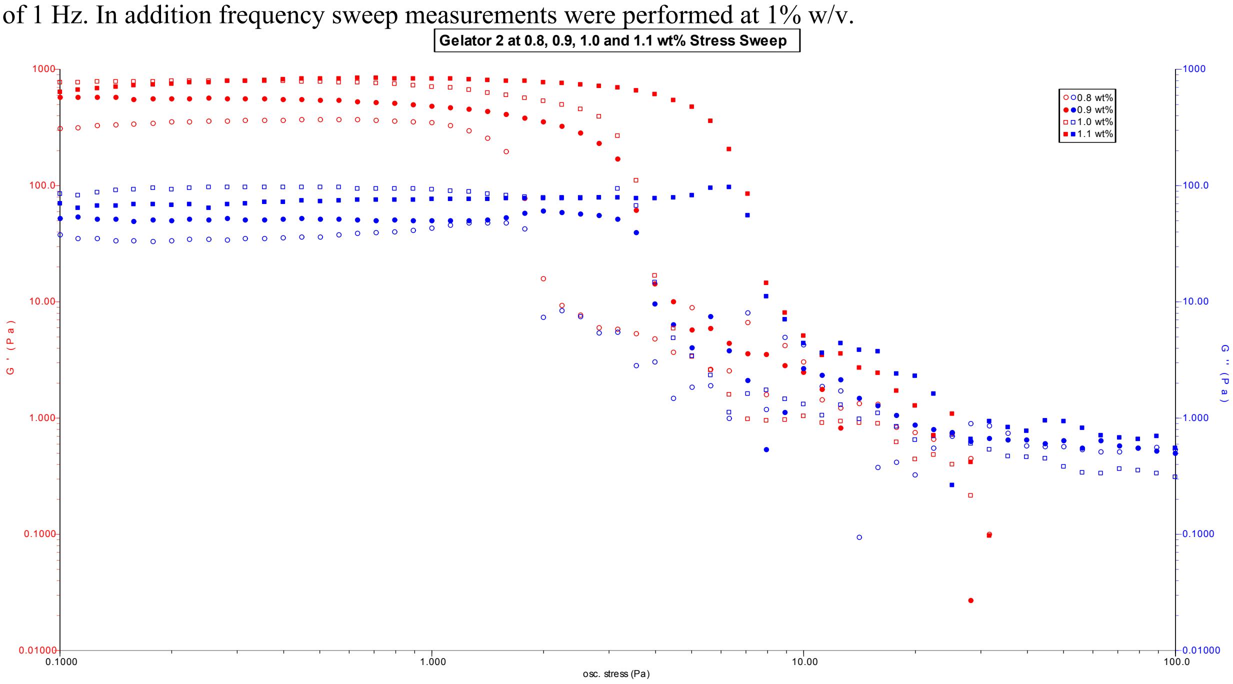

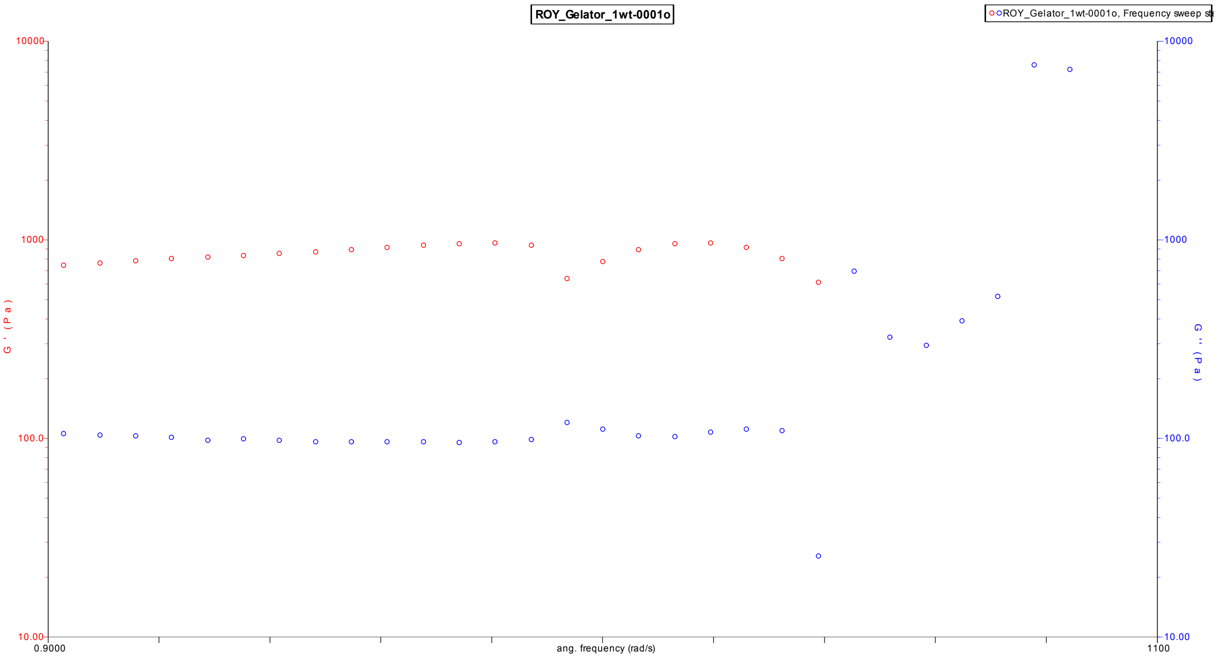

Structures of non-ROY-specific gelators used in control crystallizations

The control gelators **3** – **7** used in the initial screen are shown below. Compounds **3**, **4** and **6** have been reported previously as described in the main text. Compounds **5** and **7** were prepared as detailed below.

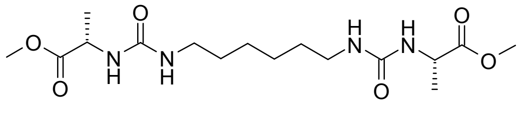

L-alanine derivative **3**

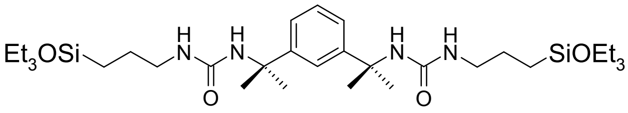

triethoxysilane derivative **6**

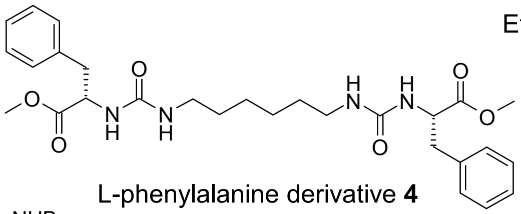

L-phenylalanine derivative **4**

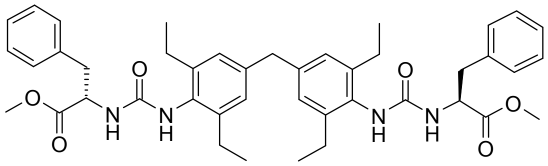

L-phenylalanine derivative **7**

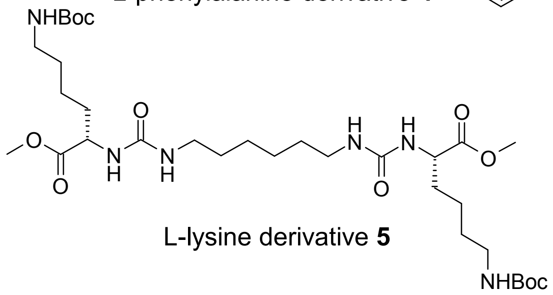

L-lysine derivative **5**

Methyl (2S)-2-[[4-[[3,5-diethyl-4-[[(1S)-1-methoxycarbonyl-3-methyl-butyl]carbamoyl amino]phenyl]methyl]-2,6-diethyl-phenyl]carbamoylamino]-4-methyl-pentanoate (**5**)

N_ε-Boc-*l*-lysine methyl ester hydrochloride (0.50 g, 1.7 mmol) was dissolved in 75 mL chloroform by the slow addition of a slight excess of triethylamine (0.182 g, 1.80 mmol). To the clear solution was added 1,6-diisocyanatohexane (0.135 g, 0.80 mmol) in 25 mL chloroform and the resulting solution was heated under reflux for 18 h. A solution was obtained which was washed with water (5 mL, twice), dried with $MgSO_4$ and filtered. The chloroform was removed under vacuum and a yellow oil obtained. The oil was partially dissolved in DCM and diethyl ether added resulting in a cloudy suspension which rapidly gelled. The mixture of solvents was reduced under vacuum and a white powder was recovered (0.39 g, 0.56 mmol, yield: 67 %) and identified as the product: ^{1}H NMR (700MHz, DMSO-d_6, J/Hz): 6.73 (1H, t, *J* 5.2, N<u>H</u>Boc), 6.14 (1H, d, *J* 8.3, CHN<u>H</u>), 5.93 (1H, t, *J* 5.2, N<u>H</u>CH_2), 4.02 (1H, dd, *J* 6.8, 13.0, C<u>H</u>), 3.14 (3H, s, OCH_3), 2.91 (2H, dd, *J* 8.0, 13.2 , NHC<u>H_2</u>), 2.82 (2H, dd, *J* 6.8, 13.0, C<u>H_2</u>NHBoc), 1.55 (2H, m, CHC<u>H_2</u>),1.54 (9H, s, C(C<u>H_3</u>)$_3$), 1.46 (2H, m, -CH_2-), 1.32 (2H, m, -CH_2-), 1.30 (2H, m, $NHCH_2C$<u>H_2</u>), 1.19 (2H, m,$CH_2CH_2CH_2$). m/z (ES$^+$ MS): 711,4 ([M+Na$^+$], 100%), 689,4 ([M], 20%). Anal. Cal for: $C_{32}H_{61}N_6O_{10}$: C 55,80; H 8,78; N 12,20. Found: C 54,83; H 9.00; N 12.01 %.

Methyl (2S)-2-[[4-[[4-[[(1S)-1-benzyl-2-methoxy-2-oxo-ethyl]carbamoylamino]-3,5-diethyl-phenyl]methyl]-2,6-diethyl-phenyl]carbamoylamino]-3-phenyl-propanoate (**7**)

L-Phenylalanine methyl ester hydrochloride (0.50 g, 2.32 mmol) was dissolved in 20 mL of chloroform and an excess of triethylamine added. 4,4′-methylenebiz(2,6-diethylphenylisocyanate) (0.42 g, 1.16 mmol) in 20 mL chloroform solution was added dropwise and the reaction was then left stirring at 70°C for 18 h. The solution was washed with water and the product isolated by removing the solvent on a rotary evaporator. Compound **7** was isolated as a white powder (0.42 g, 0.58 mmol, 50 %): ^{1}H NMR (500 MHz, DMSO-d_6, *J*/Hz): 7.54 (2H, s, ArNH), 7.33-7.24 (10 H, m, ArH), 6.90 (4H, s, ArH), 6.49 (2H, s, CHNH), 4.49-4.44 (2H, m CH), 3.79 (2H, s, ArCH_2Ar), 3.61 (6H, s, OCH_3), 2.99 (4H, d, *J* 9.8, CHCH_2Ar), 2.44-2.39 (8H, m, ArCH_2), 1.03 (12H, t, *J* 7.4, CH_2CH_3). ^{13}C NMR{^{1}H} (126 MHz, DMSO-d_6, *J*/Hz): 173.5 (s, COO), 156.7 (s, NHCO), 142.5 (s, ArC), 139.9 (s, ArC), 145.0 (s, ArC), 137.8 (s, ArC), 128.9 (s, ArC), 127.2 (s, ArC), 126.9 (s, ArC), 54.7 (s, CH), 52.4 (s, OCH3), 41.4 (s, ArCH_2Ar), 25.1 (s, ArCH_2CH_3), 15.4 (s, ArCH_2CH_3). m/z (ES^{+}-MS): 102.2 (Et_3N+H]$^{+}$, 100%, 822.6 ([M+Et_3N]$^{+}$, 42 %), 823.6 ([M+Et_3N]$^{+}$, 30%). Anal. Calc. for $C_{43}H_{52}N_4O_6$: C, 71.64; H, 7.27; N, 7.77 %. Found: C, 71.29; H, 7.30; N, 7.70 %.

Table S1 Crystallisation and polymorphism of ROY crystallised from different gelators and solution over time. First screening experiments.

Gelator	ROY mg/ml toluene	Crystal form observed at time interval				
		24 hours	48 hours	96 hours	2 weeks	>1 month
**2**	100	–	–	Y	Y	Y
	100	–	–	R	R	R
	100	–	R	R	R	R
	100	–	–	R	R	R
	100	–	–	R	R	R
	50	–	–	R	R	R
	150	–	R	R	R	R
	200	–	Y	Y	Y	Y
**3**	100	Y	Y	Y	Y	Y
	100	Y	Y	Y	Y	Y
	100	Y	Y	Y	Y	Y
	100	-	R	R	R	R
	100	Y	Y	Y	Y	Y
	50	-	Y	Y	Y	Y
	150	-	Y	R+Y	R+Y	R+Y
	200	-	R	R	R	R
**4**	100	Y	Y	Y	Y	Y
	100	Y	R+Y	R+Y	R+Y	R+Y
	100	Y	Y	Y	Y	Y
	100	Y	Y	Y	Y	Y
	100	R+Y	R+Y	Y	Y	Y
	50	-	-	-	-	Y
	150	Y	Y	Y	Y	Y
	200	R+Y	R+Y	R+Y	R+Y	R+Y
**6**	100	Y	Y	Y	Y	Y
	100	Y	Y	Y	Y	Y
	100	Y	Y	Y	Y	Y
	100	Y	Y	Y	Y	Y
	100	Y	Y	Y	Y	Y
	50	-	-	-	Y	Y
	150	Y	Y	Y	Y	Y
	200	Y	Y	Y	Y	Y
Solution (no gelator)	100	-	Y	Y	Y	Y
	100	-	Y	Y	Y	Y
	100	-	Y	Y	Y	Y
	100	-	Y	Y	Y	Y
	100	-	Y	Y	Y	Y
	50	-	-	-	Y	Y
	150	Y	Y	Y	Y	Y
	200	Y	Y	Y	Y	Y
Solution saturated with **2** at 20°C	100	Y	Y	Y	Y	Y
	100	-	-	-	Y	Y
	100	R	R	R	R	R
	100	-	Y	Y	Y	Y
	100	-	R	R	R	R
	50	-	-	-	-	Y
	150	Y	Y	Y	Y	Y
	200	-	Y	Y	Y	Y

Y and R refer to the crystal form of ROY obtained (S. Chen, I. A. Guzei, L. Yu, *J. Am. Chem. Soc.* **2005**, *127*, 9881) . R+Y indicates concomitant polymorphism. All gelators used at 1% w/v in toluene except **3** which was used at 1.5 % w/v.

Table S2 ROY crystallisation experiments of using designer and control gelators as well as from toluene solution. Second (optimised) screening experiments. In a typical experiment, 1 mL toulene was added to gelator (10 mg) and ROY (100 mg) in a vial, sealed and heated to 140 ^{0}C in a DrySyn Multi-reaction station (Figure S3) until all solids had completely dissolved and then immediately placed in an oven at 120 ^{0}C. The solutions were cooled to 50 ^{0}C over 23 h and then to room temperature over a further 10h. Crystallisation generally took place over several hours to weeks. Pictures of the vials are shown in Figure S4.

Gelator	ROY mg/mL toluene	Crystal form observed at time interval					
		24 hours	48 hours	72 hours	96 hours	2 weeks	>1 month
**2**	100	–	–	R	R	R	R
	100	–	–	Y	Y	Y	Y
	100	–	–	R	R	R	R
	100	–	R	R	R	R	R
	100	–	–	R	R	R	R
	100		–	R	R	R	R
	100	–	R	R	R	R	R
	50	–	–	R	R	R	R
	150	–	R	R	R	R	R
	200	–	Y	Y	Y	Y	Y
**7**	100	Y	Y	Y	Y	Y	Y
	100	R+Y	R+Y	Y	Y	Y	Y
	100	Y	Y	Y	Y	Y	Y
	100	Y	Y	Y	Y	Y	Y
	100	Y	Y	Y	Y	Y	Y
	100	Y	Y	Y	Y	Y	Y
	100	–	R	R+Y	R+Y	R+Y	R+Y
	50	–	–	–	–	Y	Y
	150	R+Y	R+Y	R+Y	R+Y	R+Y	R+Y
	200	Y	Y	Y	Y	Y	Y
Solution (no gelator)	100	–	Y	Y	Y	Y	Y
	100	–	Y	Y	Y	Y	Y
	100	–	Y	Y	Y	Y	Y
	100	–	Y	Y	Y	Y	Y
	100	–	Y	Y	Y	Y	Y
	100	–	Y	Y	Y	Y	Y
	100	–	Y	Y	Y	Y	Y
	50	–	–	–	–	Y	Y
	150	Y	Y	Y	Y	Y	Y
	200	Y	Y	Y	Y	Y	Y

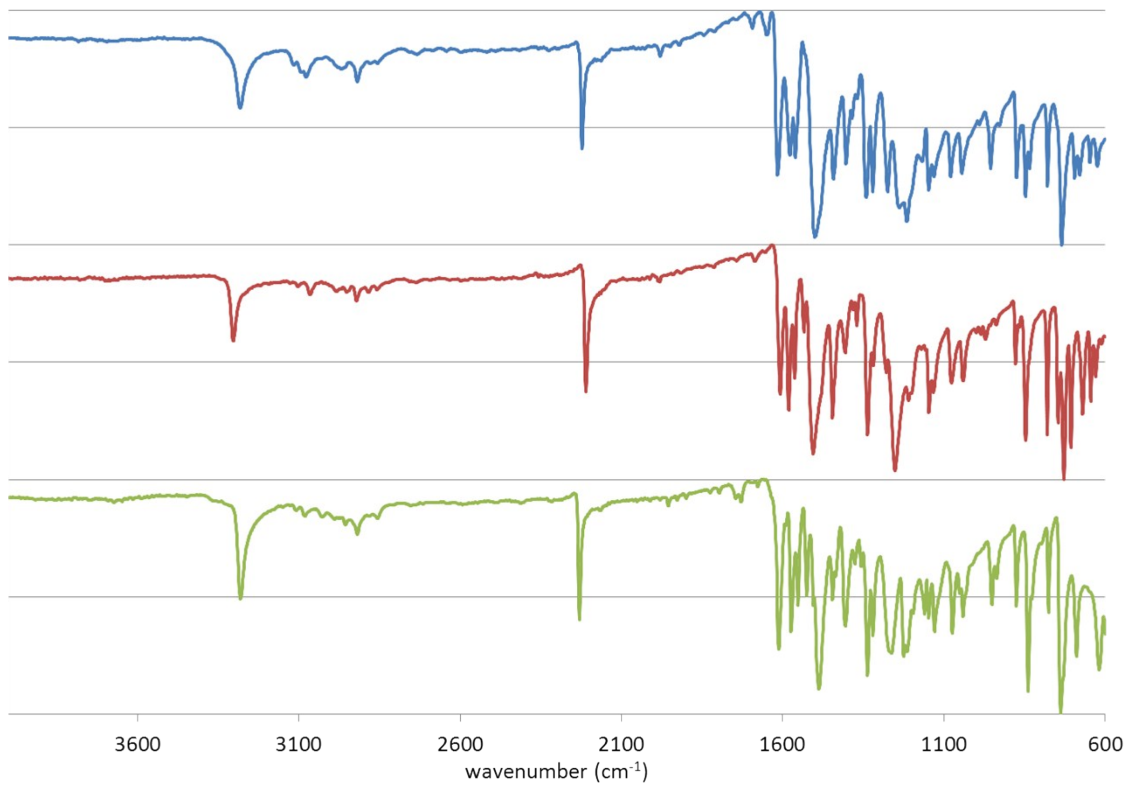

Figure S1 Typical IR spectra for ON (blue), R (red) and Y (green) polymorphs of ROY grown from gels**.**

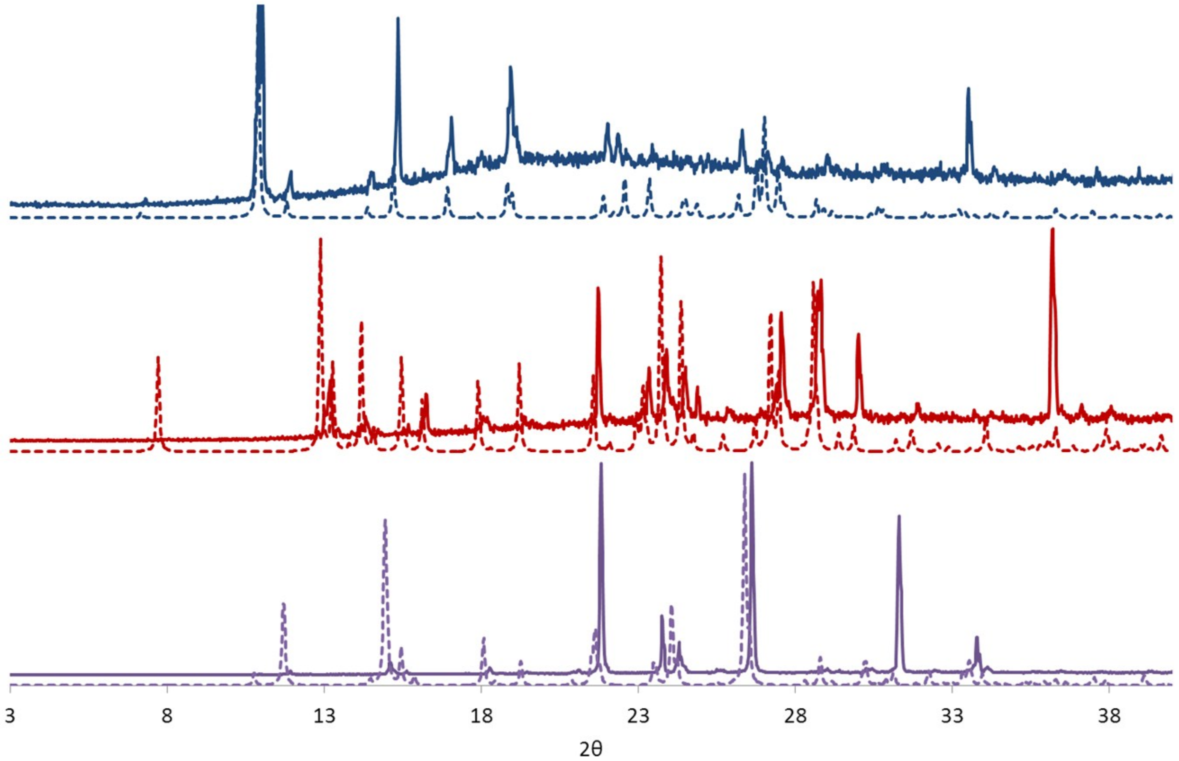

Figure S2 Assignment of crystal forms of ROY based on comparison of experimental (unbroken lines) and calculated (dotted lines) XRPD patterns: ON form, QAXMEH (blue), R form, QAXMEH02 (red), Y form, QAXMEH01 (purple) - L. Yu, G. A. Stephenson, C. A. Mitchell, C. A. Bunnell, S. V. Snorek, J. J. Bowyer, T. B. Borchardt, J. G. Stowell, S. R. Byrn, *J. Am. Chem. Soc.* **2000**, *122*, 585.

Table S3 Crystallization in ethanol

Crystallisation experiments of ROY were also performed in ethanol with **2** and **7**. In most cases, the R form was obtained and solution control crystallisation without the gelator resulted in orange coloured powder and gel (OR/G) at very low (25 mg) and high concentration (150 mg and above) of ROY.

Gelator	ROY mg/mL ethanol	Crystal form observed at time interval					
		24 hours	48 hours	72 hours	96 hours	2 weeks	>1 month
**2**	25	R	R	R	R	R	R
	25	R	R	R	R	R	R
	50	R	R	R	R	R	R
	50	R	R	R	R	R	R
	50	R	R	R	R	R	R
	100	R	R	R	R	R	R
	100	R	R	R	R	R	R
	100	R	R	R	R	R	R
	100	R	R	R	R	R	R
	150	R	R	R	R	R	R
**7**	25	R	R	R	R	R	R
	25	R	R	R	R	R	R
	50	R	R	R	R	R	R
	50	R	R	R	R	R	R
	50	R	R	R	R	R	R
	100	R	R	R	R	R	R
	100	R	R	R	R	R	R
	100	R	R	R	R	R	R
	100	R	R	R	R	R	R
	150	R	R	R	R	R	R
Solution (no gelator)	25	R	R	R	R	R	R
	25	R	R	R	R	R	R
	50	R	R	R	R	R	R
	50	R	R	R	R	R	R
	50	OR/G	OR/G	OR/G	OR/G	OR/G	OR/G
	100	R	R	R	R	R	R
	100	R	R	R	R	R	R
	100	OR/G	OR/G	OR/G	OR/G	OR/G	OR/G
	100	OR/G	OR/G	OR/G	OR/G	OR/G	OR/G
	150	R	R	R	R	R	R

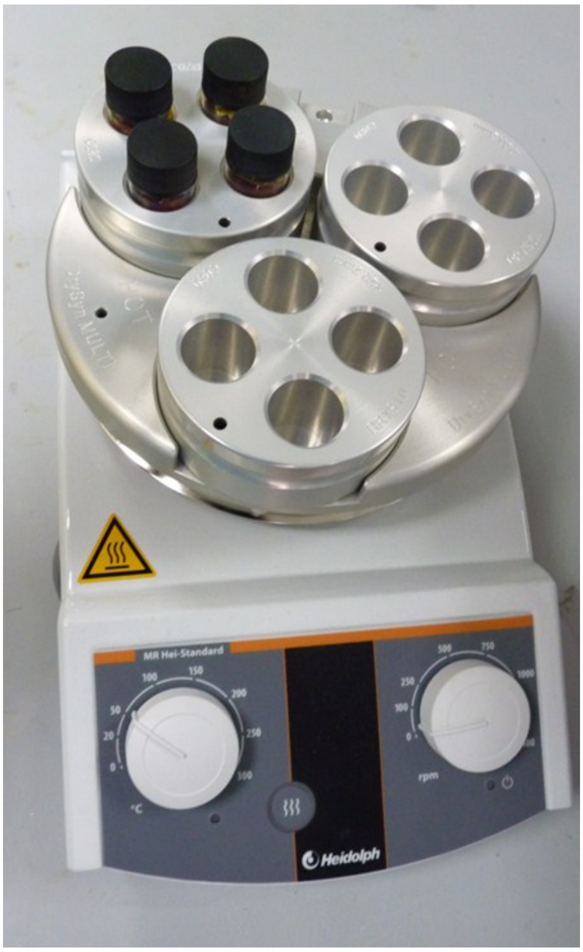

Figure S3. DrySyn Multi-reaction station used for controlled crystallization experiments.

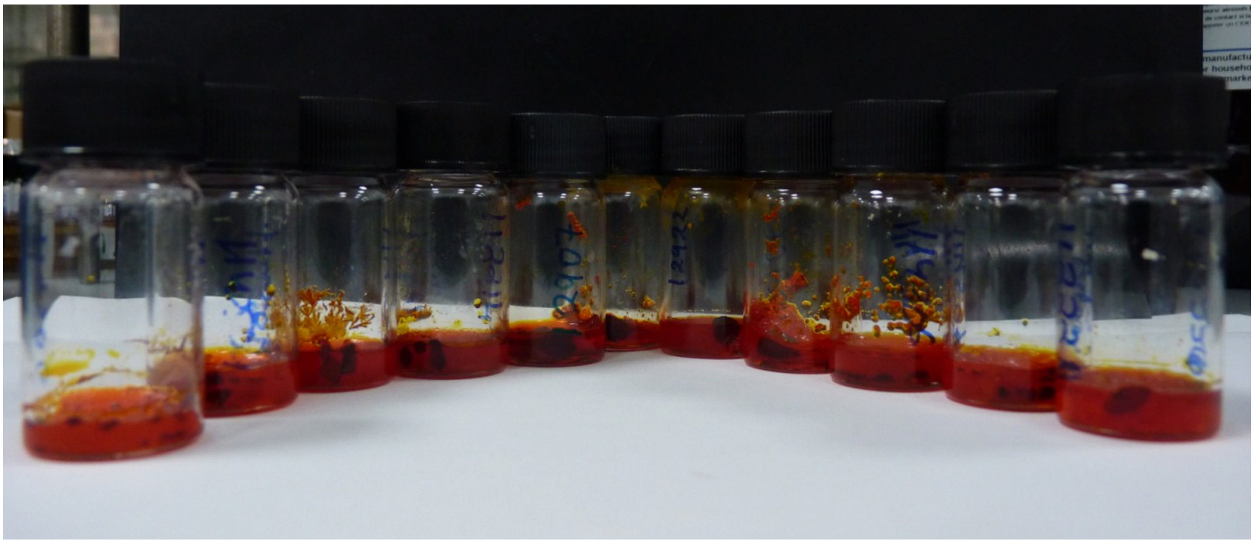

Figure S4 Crystallization of ROY in gels of **2**. In a typical experiment, 1 mL toulene was added to gelator (10 mg) and ROY (100 mg) in a vial, sealed and heated to 140 ^{0}C in a DrySyn Multi-reaction station until all solids had completely dissolved. The vials were removed from the reaction station and allowed to cool to room temperature.

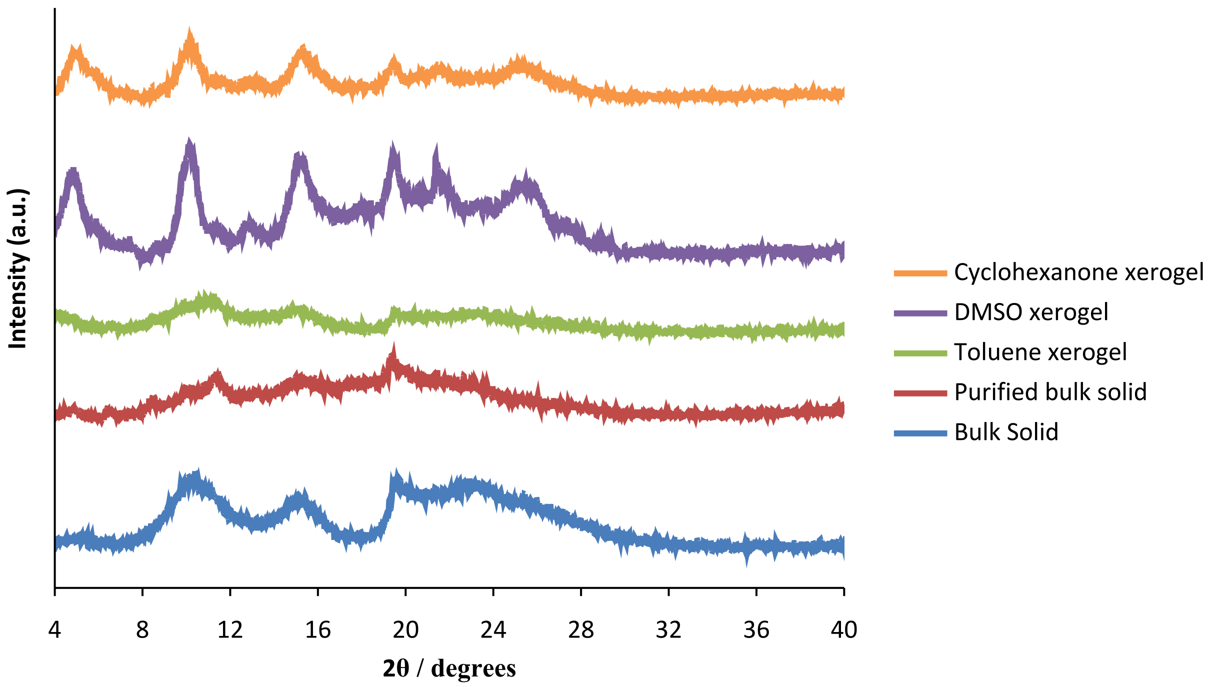

Figure S5 Vertically offset XRPD data for gelator **2**. The bulk solid was prepared from chloroform and then purified by washing the compound with copious quantities of chloroform (see experimental).

Conformational searches

A search for conformers of **2** was performed using a low-mode conformational search (I. Kolossváry, W. C. Guida, J. *Am. Chem. Soc.* 1996, **118**, 5011-5019.) method, as implemented in MacroModel (Schrodinger LLC, New York, NY, MacroModel, V9.0, 2011.). This is a mode-following algorithm – a starting molecular geometry is perturbed along one or a combination its calculated normal modes before re-minimising. The OPLS-AA (W. L. Jorgensen and J. Tirado-Rives, *J. Am. Chem. Soc*., 1988, **110**, 1657–1666) force field was used in these searches.

Minimum and maximum move distances of 3 and 6 Å were applied and 32,000 search steps were performed. A gradient of < 0.05 kJ mol^{-1} $Å^{-1}$ was set as a criterion for convergence of geometry optimisations. All conformations within a 50 kJ mol^{-1} window of the global minimum were saved from the initial search, both to keep all conformers that might be relevant to crystal packing and to allow for significant inaccuracies of the force field. Duplicate molecular geometries were identified and removed first using an all-atom RMS deviation of atomic positions (within Macromodel), with a 0.05 Å tolerance, followed by clustering based on selected dihedral angles (performed using in-house software), with tolerances of 5 RMS and 10 maximum dihedral angle difference (in degrees) to identify duplicate conformers.

Specific searches for anti-anti conformer were performed, starting with both urea groups in the anti-anti conformation, again performing 32,000 search steps, but allowing higher energy conformers to be saved.

All conformers were re-optimised using density functional theory, with an empirical correction for dispersion energies (B3LYP/6-31G** with the GD3BJ (S. Grimme, S. Ehrlich and L. Goerigk, *J. Comp. Chem*. 2011, **32**, 1456-65) dispersion correction) using Gaussian09 (Gaussian 09, Revision D.01, M. J. Frisch, G. W. Trucks, H. B. Schlegel, G. E. Scuseria, M. A. Robb, J. R. Cheeseman, G. Scalmani, V. Barone, B. Mennucci, G. A. Petersson, H. Nakatsuji, M. Caricato, X. Li, H. P. Hratchian, A. F. Izmaylov, J. loino, G. Zheng, J. L. Sonnenberg, M. Hada, M. Ehara, K. Toyota, R. Fukuda, J. Hasegawa, M. Ishida, T. Nakajima, Y. Honda, O. Kitao, H. Nakai, T. Vreven, J. A. Montgomery, Jr., J. E. Peralta, F. Ogliaro, M. Bearpark, J. J. Heyd, E. Brothers, K. N. Kudin, V. N. Staroverov, T. Keith, R. Kobayashi, J. Normand, K. Raghavachari, A. Rendell, J. C. Burant, S. S. Iyengar, J. Tomasi, M. Cossi, N. Rega, J. M. Millam, M. Klene, J. E. Knox, J. B. Cross, V. Bakken, C. Adamo, J. Jaramillo, R. Gomperts, R. E. Stratmann, O. Yazyev, A. J. Austin, R. Cammi, C. Pomelli, J. W. Ochterski, R. L. Martin, K. Morokuma, V. G. Zakrzewski, G. A. Voth, P. Salvador, J. J. Dannenberg, S. Dapprich, A. D. Daniels, O. Farkas, J. B. Foresman, J. V. Ortiz, J. Cioslowski and D. J. Fox, Gaussian, Inc., Wallingford CT, 2013.).

Connolly surface areas were calculated using a probe radius of 1.4 Å.

Conformers were re-ranked by adding a surface area term to the calculated (DFT-D) energy, as suggested by Thompson and Day (H. P. G. Thompson and G. M. Day, *Chem. Sci.*, 2014, **5**, 3173):

$$\Delta E_{conf,biased} = \Delta E_{DFT-D} + 0.75\ \mathrm{kJ\ mol^{-1}\ Å^{-2}} A_{Connolly}$$

Where ΔE_{DFT-D} is the relative energy, calculated with respect to the lowest energy conformer, $A_{Connolly}$ is the Connolly surface area and the 0.75 $kJ\ mol^{-1}\ Å^{-2}$ factor approximates the intermolecular stabilisation gained by an increase in molecular surface area.

Crystal structure prediction (CSP)

Trial crystal structures were generated in 16 space groups (*i.e.* $P1$, $P\bar{1}$, $P2_1$, $P2_1/c$, $P2_12_12$, $P2_12_12_1$, $Pna2_1$, $Pca2_1$, *Pbca*, *Pbcn*, *C*2/*c*, *Cc*, *C*2, *Pc* $P4_12_12$, and $P4_32_12$) using the *CrystalPredictor* program (Karamertzanis, P. G.; Pantelides, C. C., *J. Comput. Chem.* 2005**,** *26*, 304-324), which generates structures using a low-discrepancy sequence to sample the degrees of freedom that define the crystal structure (unit cell parameters, molecular positions and orientations). 200,000 crystal structures were generated and energy minimised per conformer, using the W99 force field and atomic partial charges fitted to the B3LYP/6-31G** molecular electrostatic potential. Molecular geometries were held rigid at this stage.

The lowest energy crystal structures were re-optimised allowing flexibility around all exo-cyclic torsion angles and bond angles using CrystlOptimizer (Kazantsev, A. V.; Karamertzanis, P. G.; Adjiman, C. S.; Pantelides, C. C., *J. Chem. Theory Comput.* **2011,** *7*, 1998-2016), which used the DFT energy model for the molecular energy, along with the W99 force field for intermolecular interactions, and atomic multipoles up to hexadecapole on each atom for intermolecular electrostatic interactions. Multipoles were derived from a distributed multipole analysis (Stone, A. J.; Alderton, M., *Mol. Phys.* **1985,** *56*, 1047-1064). All intermolecular interactions were summed to a 30 Å cutoff, apart from charge-charge, charge-dipole and dipole-dipole interactions, for which Ewald summation was applied.

PXRD simulations

Powder X-ray diffraction patterns were simulated form the lowest energy predicted crystal structures using Mercury (C. F. Macrae, I. J. Bruno, J. A. Chisholm, P. R. Edgington, P. McCabe, E. Pidcock, L. Rodriguez-Monge, R. Taylor, J. van de Streek and P. A. Wood, *J. Appl. Cryst*., **41**, 466-470, 2008). Preferred orientation effects on the intensities was modelled, assuming needle-like growth along the direction of hydrogen bond chains and a March-Dollase parameter of 1.5.

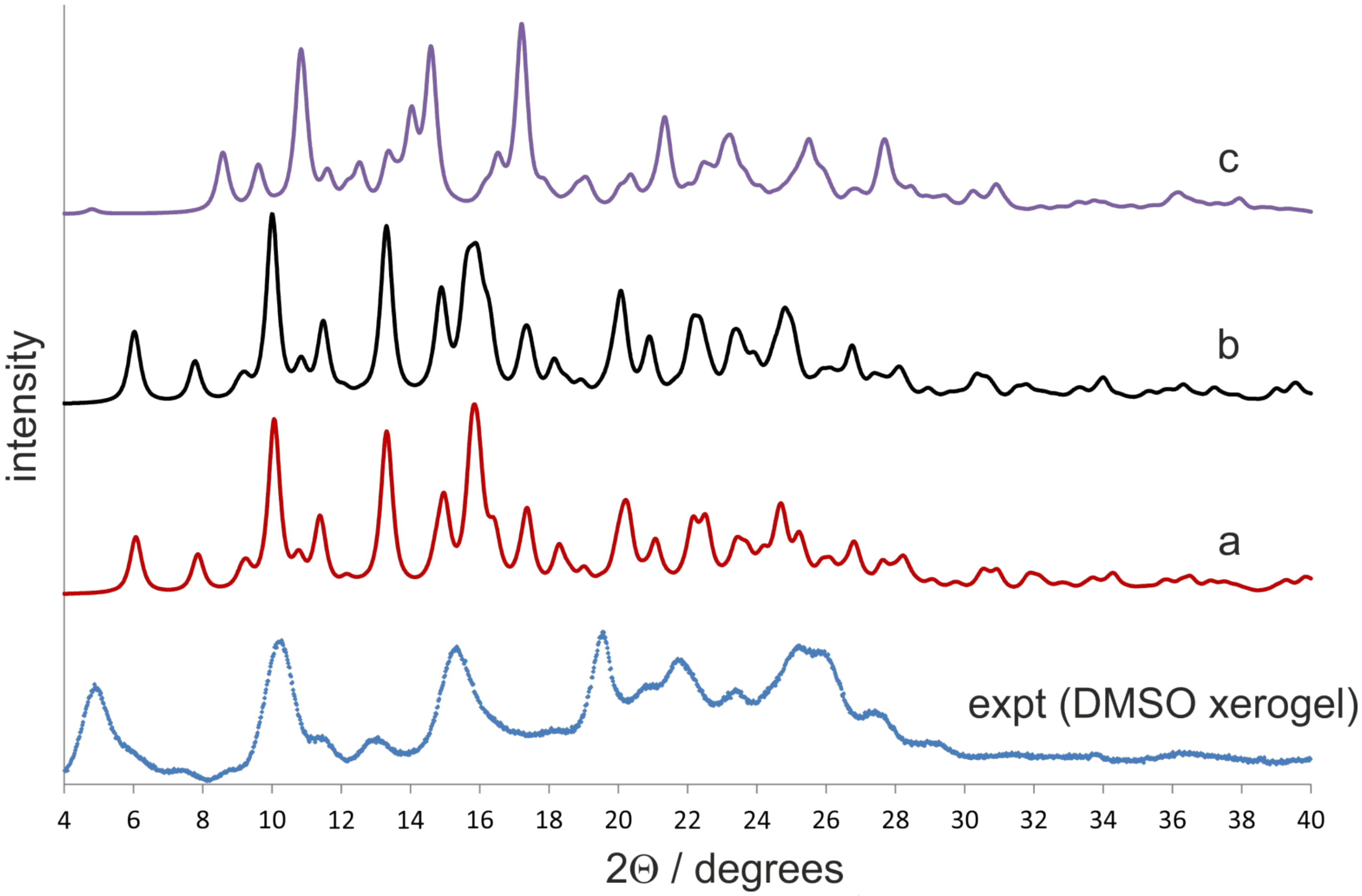

Figure S6. Simulated powder X-ray diffraction from the a) lowest energy, b) 2nd lowest energy and c)_third lowest energy predicted crystal structures from the extended conformers of **2**. These are compared to the DMSO xerogel.
